# Male-Biased Sexual Size Dimorphism, Resource Defense Polygyny, and Multiple Paternity in the Emei Moustache Toad (*Leptobrachium boringii*)

**DOI:** 10.1371/journal.pone.0067502

**Published:** 2013-06-28

**Authors:** Cameron M. Hudson, Jinzhong Fu

**Affiliations:** Department of Integrative Biology, University of Guelph, Guelph, Ontario, Canada; Monash University, Australia

## Abstract

We tested the hypotheses that the Emei moustache toad (*Leptobrachium boringii)* exhibits resource defense polygyny and that combat led to the evolution of male-biased sexual size dimorphism. Between February and March of 2011 and 2012, 26 female and 55 male *L. boringii* from Mount Emei UNESCO World Heritage Site, Sichuan, China, were observed throughout the breeding season. Prior to the breeding season, males grow 10–16 keratinized maxillary nuptial spines, which fall off once the season has ended. Throughout this time, males construct and defend aquatic nests where they produce advertisement calls to attract females. In a natural setting, we documented 14 cases involving a total of 22 males where males used their moustaches for aggressive interaction, and nest takeover was observed on seven occasions. Males were also observed to possess injuries resulting from combat. Genetic analysis using microsatellite DNA markers revealed several cases of multiple paternity, both within nest and within clutch. This observation indicated that some alternative male reproductive strategy, such as satellite behaviour, is occurring, which may have led to the multiple paternity. Larger males were observed to mate more frequently, and in multiple nests, suggesting that females are selecting for larger males, or that larger males are more capable of defending high quality territories.

## Introduction

Across a wide variety of species, natural selection and sexual selection has produced marked differences between the sexes and as a consequence, sexual dimorphism has shaped the life history and evolution of many organisms. The differences are predicted to be adaptations of males and females to their disparate reproductive roles, however, the exact mechanism by which these adaptations came to exist depends on the species in question and its mating system. Sexual size dimorphism (SSD) can be found throughout the animal kingdom. Although the majority of taxa exhibit female-biased SSD [1], there are many species which exhibit male-biased SSD, most notably, birds and mammals. The advantages to large male body size have been listed in [1] as: (1) dominance in contests of strength, (2) greater endurance, (3) female preference for large males, and (4) higher success in sperm competition. Two mechanisms, resource defence polygyny [2], and parental investment [3] have been suggested to explain the evolution of male-biased SSD in animals. These hypotheses are primarily derived from studies of birds and mammals and whether they are applicable in the animal kingdom as a whole requires further research. Since male-biased SSD is the exception in animals, studying species that deviate from the norm may provide insight into the generality of these hypotheses.

Amphibians of the order Anura contain a fascinating number of diverse mating systems, which offer excellent opportunities to test these hypotheses [4]. The majority of amphibians exhibit female-biased SSD, though an estimated 10% of species have evolved male-biased SSD [5]. Advertisement calling is the primary method that anuran males use to attract mates, however some species have been observed to engage in territory defence and combat [4]. Shine [5] first hypothesized that combat between males produces selection pressure for the evolution of larger body size, as well as the development of spines and tusks as an adaptation for combat. Han and Fu [6] recently examined the correlation between SSD and several life history traits in anurans and concluded that parental care, rather than male combat, may be more important in driving male-biased SSD. Thus the significance of male combat in determining male-biased SSD and its mechanism remains unclear. To establish the role of combat in the evolution of male-biased SSD in anurans, case studies are probably the best approach to review mechanistic causative relationships.

The Emei moustache toad (*Leptobrachium boringii*, formerly *Vibrissaphora boringiae*; [7]) represents an excellent model organism for studying the relationship between male weapon development and the evolution of male-biased SSD in anurans. The species exhibits male-biased SSD, hypertrophied forearms and conspicuous keratinized nuptial spines that grow on the upper lip of males during the breeding season. Each male can grow between 10–16 sharp, conical black spines, which re-grow if broken during this time. The spines are 3–5 mm in length, and are oriented away from the snout [8]. The breeding season for *L. boringii* typically lasts 2–3 weeks, and therefore they can be classified as explosive breeders according to the definition of Wells [9]; however males remain with the eggs in the site much longer. The fact that *L. boringii* is an explosive breeder can increase competition between males as well, since mating opportunities are infrequent and the breeding aggregations are densely populated. Males also demonstrate signs of parental investment through pre-copulatory nest construction behaviours [10], [11], [12]. Following an initial reproductive event, a male will continue to call in an attempt to attract and mate with additional females. During this time, males remain with the eggs and have been shown to lose roughly 7.3% of their body mass throughout the season, suggesting that larger males will be able to remain in the aggregation for longer periods of time [13]. In previous field studies, the number of egg masses per nest varied from zero to ten, and this implies a disparity in mating success between different males in the population [13]. Through examining *L. boringii* populations across several years, Zheng *et al*., [13] have reported a male to female sex ratio which varies from 1∶ 1.5 to 1∶ 2.5 however, females typically leave the breeding aggregation promptly after mating, while males remain for over a month, and thus the operational sex ratio at any time point is highly male biased. A genetic analysis of microsatellite DNA diversity in *L. boringii* eggs [13] reported four cases in which a single egg mass was sired by at least two males, and two cases where a nest had egg masses sired by different males. This suggests that the resident male in a nest may not be the original owner, or that an alternative reproductive strategy is occurring in which a secondary male is fertilising some of the eggs in a clutch (i.e. clutch piracy; see [14]). These biological traits allow us to test hypotheses related male-biased SSD.

Male combat and resource defense polygyny have been suggested as evolutionary mechanisms that resulted in male-biased SSD in *L. boringii* [7], [13], [15], [16], [17]. In this study we tested this hypothesis by: (1) providing evidence for male combat and multiple paternity and, (2) linking male body size to success in combat or reproduction. Combat was expected to occur early in the breeding season, in which males would use their keratinized nuptial spines as weapons against other males and compete for a limited number of appropriate nest sites. Based on the finding of multiple paternity from [13], males were expected to be performing an alternative reproductive strategy via some unknown mechanism, as the amplectic behaviours in *L. boringii* are complex. In this study we aim to provide evidence supporting the hypothesis that male-biased SSD in *L. boringii* is the result of selection on male body size from male combat and resource defense polygyny.

## Materials and Methods

### Study Site and Sampling Procedures

We studied a population of *L. boringii* within the confines of Mount Emei UNESCO World Heritage Site, Sichuan, China (N29.567°, E103.417°, elevation 650–890 m) between February 10^th^ and March 18^th^ of 2011 and 2012. Suitable habitat was identified by the presence of *L. boringii* tadpoles from previous breeding seasons, or large, flat rocks in slow moving water, which are appropriate nest locations. We attempted to locate all adults that attended the breeding aggregation. Individuals were captured when found in the open, or located by searching under rocks with the aid of Mastercraft® underwater inspection cameras. In total, 77 *L. boringii* specimens were collected, (19 females : 43 males; 2011) (7 females : 8 males; 2012).

Captured toads were measured for seven morphological metrics (snout-vent length, mass, radioulna length, femur length, tibiofibula length, foot length and head width). Females were scored for the presence of eggs and the number of keratinized nuptial spines was counted in males. Measurements were taken using digital calipers and a set of Pesola® scales from 20 g to 100 g. *L. boringii* specimens were individually tagged with a subcutaneous Allflex® 12 mm×2.15 mm FDX-B 134.2 kHz passive integrated transponder (P.I.T. tag) for identification purposes. P.I.T. tags were scanned using an Agrident® AWR100 stick reader or with Biomark® 601 waterproof handheld readers. Toe clips were taken from the third digit from the right hind foot of each specimen and preserved it in 95% ethanol for DNA extraction and genotyping. This also served as further identification of recaptured individuals, in the event that P.I.T. tags were expelled. Wounds from P.I.T. tagging and toe clipping were sealed with Vetbond™ tissue adhesive to prevent infection. All surgical procedures were carried out with the approval of the University of Guelph Animal Care Committee, protocol number 11R016. Fieldwork was conducted with the approval of the Department of Forestry and Wildlife and the Office of Foreign Affairs of the Sichuan Provincial Government, and the Management Office of the Mt. Emei Nature Reserve.

### Tracking and Observation

To collect behavioural data on male combat, nest occupancy and reproductive success, we chose to focus on a 300 m long transect of the stream that contained the highest density of males and nest sites. Nest sites were observed daily to determine the presence of freshly laid eggs or males guarding the nest. Males captured within nests were scanned to confirm their identity. When combat was observed, videos were taken using underwater inspection cameras or with a Canon® PowerShot A590 IS digital camera from above. Videos were taken for the entirety of the aggressive interaction (approximately three minutes) or until the individuals were disturbed by the researcher’s presence. When the males disengaged and swam in opposite directions, we considered that aggressive interaction to be complete. Typically one male would remain in the nest site following the completion of combat. This male was considered to be the winner of the contest if it was recaptured within the same nest on subsequent nights. If neither male was identified in the nest on following nights the victor was undetermined. When possible, both males were captured and identified following combat. In instances where a new male was found occupying the nest of a previous male, a nest take over, and therefore combat, was assumed to have occurred between the two individuals. This assumption is based on the observation that 1) the majority males remain in the same nests, and 2) the breeding season is short and competition is intense, and therefore, the chance of a male voluntarily giving up a nest is small. At the end of the breeding season in 2011 (March 18), we collected 10–30 eggs from each egg mass (n = 25) within each nest (n = 15) for the purpose of paternity analysis. This analysis would provide data on male reproductive success, and whether males were performing alternative reproductive strategies. There were several nests (5, 9, 10 and 17) which were designated as a nest due to the presence of a male at one point during the season, but contained no eggs, thus they were not included in the paternity analysis. Eggs were preserved in 95% ethanol and stored at −20°C. As nests were numbered based on the order of their discovery, egg masses were assigned letters corresponding with the number in each nest (e.g. 1A, 2A and 2B, 3A).

### DNA Extraction, PCR Amplification, and Microsatellite DNA Genotyping

Tissue samples preserved in 95% ethanol were stored at −20°C. Each sample was digested overnight in an incubator at 37°C in a mixture of 400 µL STE buffer, 75 µL 10% SDS solution, and 20 µL of 20 mg/ml proteinase K. Following this, samples were extracted using the standard phenol/chloroform extraction method, and precipitated using a mixture of 125 µL isopropanol and 750 µL 7.5 M ammonium acetate. Finally, samples were washed with 200 µL of 95% ethanol, dried in a ThermoSavant DNA 120 SpeedVac®, resuspended in 50 µL of TE buffer and stored at −20°C. The concentration of each extracted sample was determined using a Thermo Scientific Nanodrop™ 8000 spectrophotometer, and working samples were diluted to 25 ng/µL of template DNA.

PCR amplification was conducted in 12.5 µL reaction mixtures containing 50 ng of template DNA, 1 unit of Taq DNA polymerase (TaKaRa), 1× PCR buffer (TaKaRa), 1.5 mM MgCl_2_, 0.2 mM of each dNTP, and 10 pmol of each primer. PCR cycling parameters were: 5 min at 95°C for initial denaturation, followed by 30 cycles of 95°C for 30 s, 30 s at the optimized annealing temperature (Table S1), then increased by 1°C/s to 72°C for 45 s, and a final extension step at 72°C for 5 min. The TET fluorescently labelled PCR products and TAMRA™ size standard marker (GeneScan™ 350, Applied Biosystems) were electrophoresed on 6% denaturing polyacrylamide gels at 1600 V for 3 hours. The gels were then visualized on a Hitachi® FMBIO II laser scanner and scored using MiraiBio Image Analysis v3.0.0.26 to determine the lengths of the microsatellite DNA fragments. A total of 62 adult *L. boringii* specimens and 226 eggs from 2011 were genotyped for 7 different microsatellite DNA loci [18].

### Paternity Analysis

Determining the paternity of each egg mass allowed us to quantify male reproductive success, as well as alternative reproductive behaviours and male nest occupancy. Using Cervus v3.0.3 [19], we conducted an allele frequency analysis on the genotypes from 62 adults captured in 2011, and tested each allele for Hardy-Weinberg equilibrium. All alleles were found to be in Hardy-Weinberg equilibrium after a Bonferroni correction (Table S1). Following this, all genotypes from 2011 adults and eggs were compared using the maximum likelihood parentage and siblingship inference program, Colony v2.0.1.4 [20] to determine paternity of each egg mass. Several runs were conducted with the following parameters: run length = medium, error rate for microsatellite markers = 5–10%, probability of males included in data = 90–100%, probability of females included in data = 50–100%. A non-specific maternal siblingship prior was established for the eggs in each egg mass as we are confident that all eggs in an egg mass have the same mother. From these runs, the best possible father for an egg mass was selected. The conclusions from this analysis were then compared to observational data (e.g. presence/absence of males within a nest, chronological order of male occupation to oviposition of egg masses) to determine the possibility of each predicted father. Two egg masses were considered positive controls, as mating between the parents was observed, which allowed for a measure of reliability of both the genotype data, and the program’s ability to correctly assign paternity.

### Statistical Analyses

Using body size data from captured males, we conducted a principal components analysis on the seven morphological metrics measured for all individuals. Doing this provided us with a multivariate measure of relative body size for each individual that could be used for comparison. The scores from the first principal component were taken as this measure of body size, and transformed by multiplying the values by −1, and adding five. This ensured that all values were positive and the largest males were represented by the largest values. The results from the 2011 paternity analysis were used to determine the number of matings a male received (hereafter referred to as reproductive success), and a Poisson regression was conducted to compare male body size to reproductive success. All statistical analyses were conducted using R version 2.12.2.

## Results

### Morphometrics

**Figure 1:**
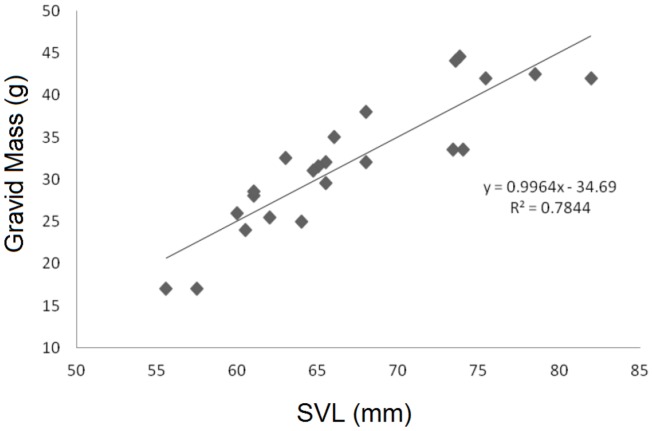
A scatter plot depicting the relationship between gravid mass and SVL for all gravid females sampled.

**Figure 2:**
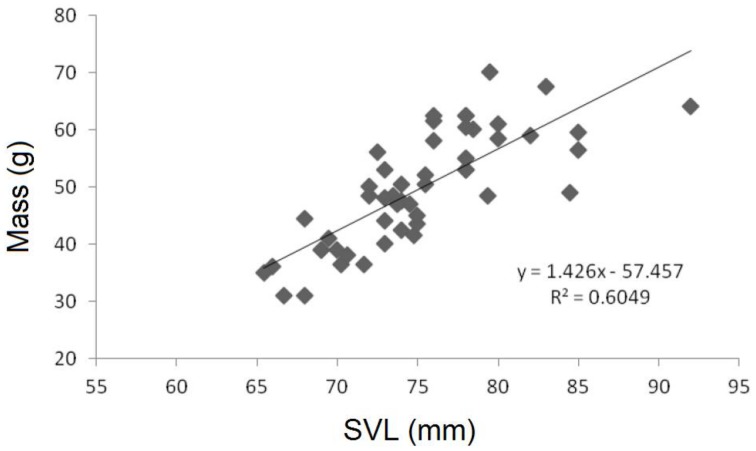
A scatter plot depicting the relationship of mass to snout-vent length in all males sampled.

Body size measurements for all individuals are shown in Table 1. Males were observed to have a mean Snout-Vent Length (SVL) of 75.2±0.748 mm (n = 51), while the mean for females was 66.5±1.31 mm (n = 26). The greatest difference between the sexes was observed in body weight, with a mean male weight of 49.8±1.37 g (n = 51) and a mean female weight of 26.6±2.39 g (n = 6), a difference of 87%. The average gravid mass of females was 31.9±1.39 (n = 23). Two females were weighed before and after oviposition and the resulting egg masses weighed 14 g and 20 g respectively; the two females were 30 g and 24.5 g post-oviposition. The smallest gravid females weighed 17 g, indicating that *L. boringii* females may become reproductively capable in the first spring after metamorphosis. Female SVL was also strongly correlated with gravid mass (r^2^ = 0.784, F = 76.42, df = 21 p<0.001 Figure 1) and male SVL was correlated to body weight (r^2^ = 0.6044, F = 74.85, df = 49, p<0.001, Figure 2). The principal components analysis for all males (n = 51) produced an accurate proxy for male body size as all axes loaded in the same direction, and the first principal component explained 62% of the variation within the model (Table 2). *L. boringii* males were shown to exhibit male-biased SSD, as shown in previous studies [13], [21], [22].

**Table 1:** Mean body size measurements of both sexes with size differential for males and females.

Measurement	Male Mean	Standard Error	N	Female Mean	Standard Error	N	Differential
Snout-vent Length (mm)	75.2	0.75	51	66.5	1.3	26	13.1%
Head Width (mm)	31.4	0.27	51	28.0	0.35	26	12.1%
Radioulnar Length (mm)	29.0	0.28	51	25.0	0.59	26	16.0%
Femur Length (mm)	33.8	0.38	51	29.6	0.61	26	14.2%
Tibiofibula Length (mm)	32.6	0.28	51	26.9	0.43	26	21.2%
Foot Length (mm)	45.7	0.48	51	39	0.42	26	17.2%
Body Weight (g)	49.8	1.37	51	26.6	2.4	6	87.2%
Gravid Mass (g)	N/A	N/A	N/A	31.9	1.6	23	N/A

**Table 2:** Proportion of variance explained by each principal component in a PCA of male body size (n = 55).

Component	1	2	3	4	5	6	7
Standard Deviation	2.09	0.90	0.87	0.62	0.53	0.47	0.44
Proportion of Variance	0.62	0.12	0.11	0.05	0.04	0.03	0.03
Cumulative Proportion	0.62	0.74	0.85	0.90	0.94	0.97	1.00

### Combat and Nest Takeover

**Figure 3:**
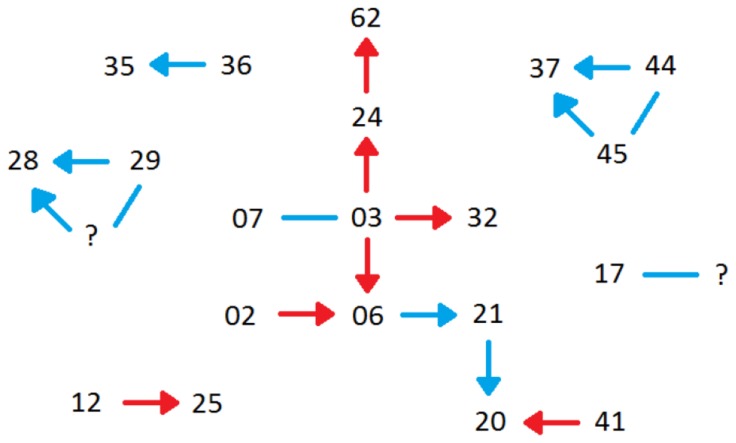
A pictographic representation of all aggressive interactions from the 2011 season. Each number corresponds to a particular male. The direction of each arrow (head) indicates the winner of combat. Blue arrows represent interactions that were directly observed and recorded, while red arrows were interactions inferred through territory takeovers. Lines with no arrow represent observed combat where the victor could not be determined.

**Figure 4:**
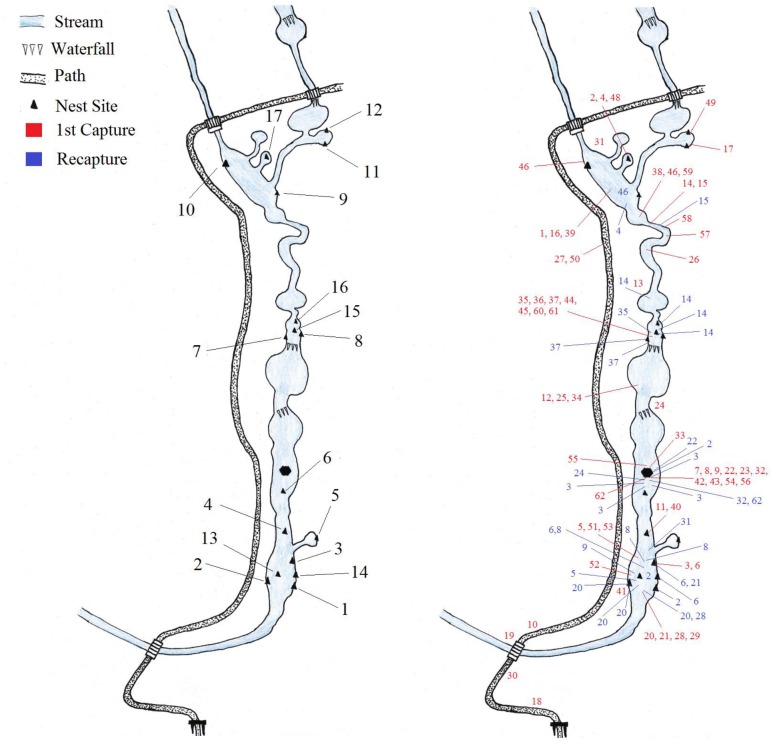
Map of Emei Mountain site A with nest locations, capture and recapture locations from all 2011 individuals.

**Figure 5:**
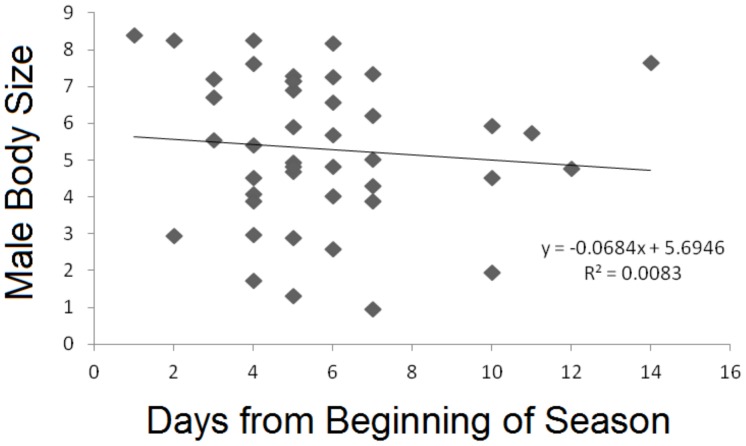
A Relationship between multivariate measure of male size (n = 43) and time of arrival to the breeding aggregation in 2011.

**Figure 6:**
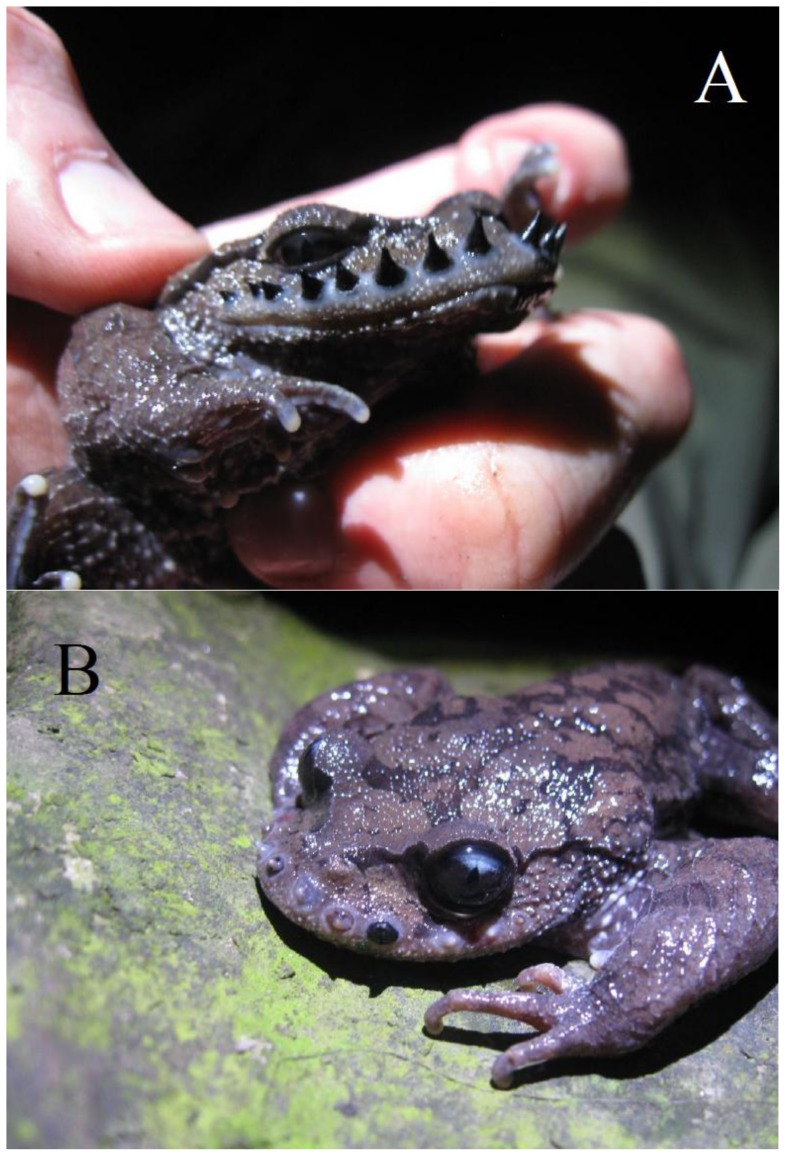
A comparison between an *L.* *boringii* male at the height of the breeding season and a male losing his nuptial spines at the end of the breeding season.

In 2011, we directly observed seven cases of male combat and recorded five of these on video. Of these seven cases, two involved three males fighting in a group over one nest site, while the other five interactions involved only two males. From the mark/recapture data however, we determined that at least 22 males were involved in aggressive interactions and territory takeovers in the study area, with a total of 14 combat events (Figure 3). Some individuals were observed fighting on multiple occasions and were found within several different nests throughout the breeding season (Figure 4). As predicted, the majority of fighting took place during the beginning of the breeding season (in 2011 fighting was first observed on February 22^nd^), and ceased once female attendance to the site had ended. The last female in 2011 was captured on March 3^rd^, no combat was observed past this date. There was no significant relationship found between male body size (n = 43) and time of arrival for the 2011 season (Figure 5). Males began to lose their nuptial spines by mid-March (Figure 6), therefore it is likely that combat ceases after a short period of intense activity. In 2011, no combat was observed after February 26^th^. During the breeding season, males were frequently found possessing wounds which were interpreted as the result of combat with conspecifics [15]. We were able to determine the winner in 13 of the aggressive interactions.

### Paternity Analysis and Reproductive Success

**Figure 7:**
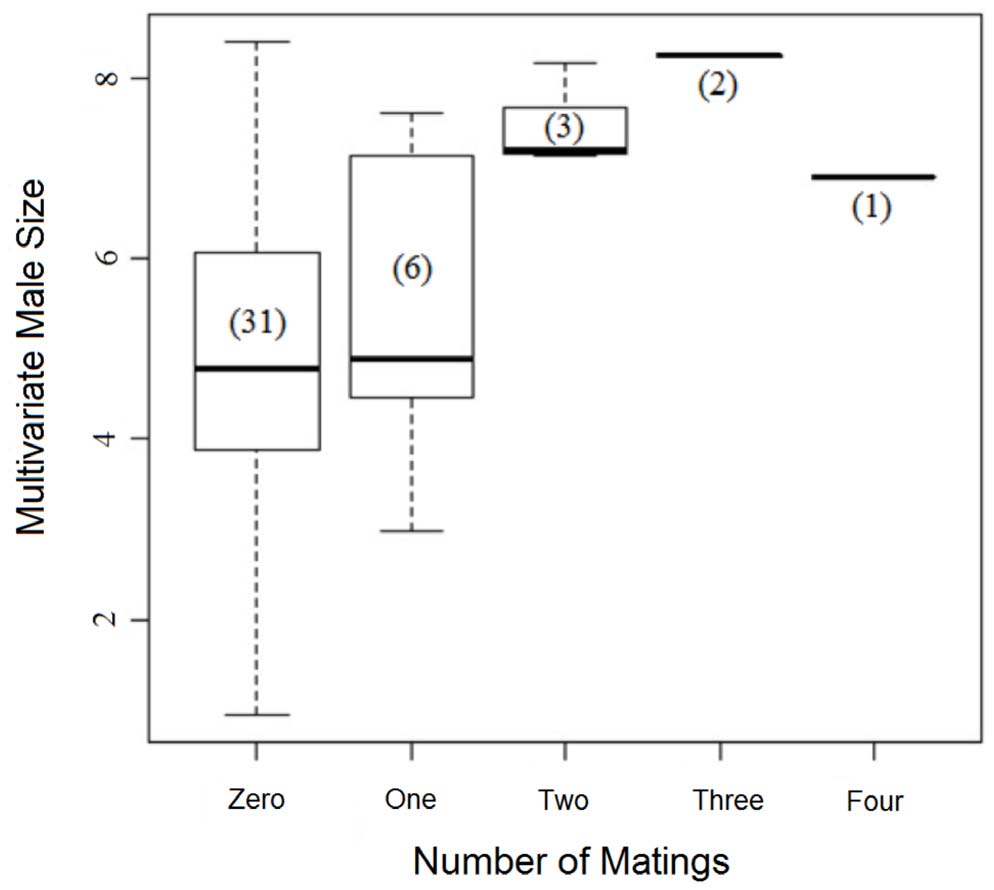
Boxplots comparing the difference between males in reproductive success in relation to their multivariate body size.

By comparing results from the paternity analysis to multivariate male body size, large males were observed to have the highest reproductive success (Figure 7). The Poisson regression model predicting the number of matings a male received based on their body size was statistically significant indicating that larger males were more likely to mate (z = 3.745, df = 42, p, 0.0001). For these data, the expected change in log(matings) for a one-unit increase in size was 0.5951. Genotype data input into Colony v. 2.0.1.4 yielded a probable father for 23 out of 25 egg masses (Table S2). The fathers of the remaining two egg masses could not be identified, therefore it is likely that the males responsible were not included in our sampling. Nests #2, 3, and 6 were shown to have egg masses from multiple males, and two egg masses (2D and 3A) showed evidence of multiple paternity, indicative of an alternative reproductive strategy (Table S2). A total of 50% of the eggs genotyped from clutch 2D and 80% of the eggs from clutch 3A were fathered by the primary male. In the case of clutch 3A, the secondary father was determined to be a male which displaced the first in a territory takeover. The secondary father for 2D could not be determined.

## Discussion

It is clear that both male combat and resource defense polygyny may have contributed to the evolution of male-biased SSD in *L. boringii*. First, *L. boringii* is male-biased in sexual size dimorphism, and exhibits male-male combat over territory with the use of keratinized maxillary nuptial spines as weapons. Unlike many territorial anurans, where combat is primarily through wrestling [4], *L. boringii* uses weapon structures in combat, and injury is a real possibility for both males involved. By injuring an opponent, a male may successfully remove them from the breeding population ensuring they will obtain more mates. This is similar to combat seen in the gladiator frogs in which males can cause permanent injury to their opponents, thereby reducing competition for females [23]. *L. boringii* appears to follow the prediction by Shine [5] that anuran species that possess weapon-like structures will engage in combat and exhibit male-biased SSD. From our observations it is clear that the primary function of the nuptial spines in *L. boringii* is as a weapon to be used in combat between males. This is further supported as the nuptial spines are only present during the breeding season, and therefore have no function in defence against predators or in foraging throughout the year. Secondly, by occupying territories, larger males achieved higher mating success. The fact that *L. boringii* males are 13.1% larger in body length, and 87.2% heavier in mass than females, as well as being one of the more extreme cases of dimorphism in the Megophridae [6] suggests that this dimorphism plays an important role in their life history. Though the breeding season for *L. boringii* is classified as explosive according to the definition of Wells [9], males have previously been shown to lose roughly 7.3% of their body mass throughout the season [13]. This suggests that larger males may be able to remain in the breeding aggregation for a longer period of time as they have more resources to expend on energetically expensive behaviours, such as calling [24], and territory defence [25].

In our previous studies, we found that the outcome of combat between males was not linked to body size or body condition [15]. Although these results may be indicative of low sample size, we hypothesize that time of arrival, or the difference in physical condition between males who arrived early to those who arrive later may be important factors in deciding victory between combatants instead of absolute body size. Males occupying territories should be expected to be more aggressive towards intruders as nest sites are a limiting resource in this mating system. Males were not observed to discriminate between their own eggs and those of another male in the nest following takeover. It is possible that once a male has mated, if he is unable to defend the nest successfully, it may be in his best interests to let a different male take over. Males who arrive first at the breeding aggregation may be the first to mate, but possibly the most frequent losers since they have already been expending energy on advertisement or combat. This may also represent a form of cuckoldry, as males who have mated early in the season can leave their clutches in the care of another individual [26].

It is possible that there has been selection on females for small size as well as males for large size, which would increase the degree of dimorphism. Across both years, the smallest gravid females captured weighed only 17 g, possibly indicating that this was their first year of reproduction. As small males are expected to be poor competitors over territory, this skews the operational sex ratio further because females can engage in reproduction at an earlier age, and this allows higher quality males to monopolize females more effectively. From the two females weighed before and after oviposition, we determined that their egg masses weighed 14 g and 20 g, therefore the clutches represented 31.8% and 44.9% of their total gravid mass. The energetic cost of egg production is high, and as suggested by Smith [27], male nest attendance may be the result of females investing so much energy in egg production that they are unable to care for the clutch. Females may also be selecting a male based on the quality of his nest or the presence of eggs [28], [29], rather than the quality of the male himself. Males who own nests or possess eggs from previous matings should be high quality mates, as they must compete with others in the population to gain and hold these resources. If this is true, it may also explain why males do not remove egg masses from previous nest occupants following takeover. If possible, future research on female choice in *L. boringii* should be conducted in the field using radio telemetry to track female movements or in a lab setting where females are given a choice between multiple males and nests, both of variable sizes.

From this study we have observed several cases of multiple paternity, both within clutch and within nest. Multiple paternity appears to be the result of nest takeover as well as some unknown alternative reproductive strategy such as satellite behaviour or clutch piracy. The results from both the tracking observations and paternity analysis confirm that multiple paternity can occur both within nest and within clutch. Originally reported by Zheng *et al*., [13], several *L. boringii* egg masses were shown to possess greater than four alleles per microsatellite loci sequenced, or more than two alleles that did not belong to the resident male captured. The first observation indicates that there are multiple fathers mating with the same female, while the second implies multiple males sequentially occupied the nest. Nest takeover often results in multiple egg masses sired by different fathers, in that different owners of the nest have successfully mated. The last male will then remain in the nest with egg masses sired by multiple males unless he is evicted by another rival. There were scenarios in which a male was occupying a nest that did not contain any eggs that he sired, however this likely represents a male attempting to attract a mate following nest takeover. It was not determined whether a male will remain with the eggs in a natural setting once the females have abandoned the breeding aggregation, if none of the clutches belonged to him. In one case (Nest #3), a male was discovered with an egg mass on Feb 21^st^ and by Feb 22^nd^ he had been replaced by a second male, and a second egg mass was present in the nest. Genotyping of the eggs showed that the first egg mass had genetic contributions from both males, while the second belonged to the second male alone. It is possible that the second male attempted to fertilize the existing clutch following takeover, but due to the time delay between the deposition of the first clutch and nest takeover, which would likely make the eggs unviable for fertilization, this implies that the second male was present earlier. The evidence of multiple paternity in this, as well as other clutches indicates that an alternative strategy such as satellite behaviour, clutch piracy, or a combination thereof is occurring at the Mount Emei site, as suggested by Zheng *et al*. [30]. Despite several years of research by multiple research groups, this behaviour has not been observed in the wild or in a lab setting. To further understand the evolution of male-biased SSD in *L. boringii* we plan to continue observations on paternal care, male combat and cuckoldry, as well as conduct experiments on female preference and attendance within the breeding aggregation.

## Supporting Information

Table S1 Characterization of *L. boringii* microsatellite primers and tests for Hardy-Weinberg equilibrium. * = Significant P-value, deviating from Hardy-Weinberg equilibrium, NS = Non-significant following Bonferroni Correction
(DOCX)

Table S2 Genotypes of fathers and eggs from each egg mass. Alleles in bold do not belong to the primary father and represent maternal contribution, or genetic contribution from an unknown male. Alleles that are underlined are shared with putative secondary fathers that were located in the nest. Egg masses with a * display multiple paternity.
(DOCX)

## References

[1] Andersson M (1994) Sexual Selection. Princeton University Press, Princeton, New Jersey, U.S.A.

[2] Emlen ST, Oring LW (1977) Ecology, sexual selection, and the evolution of mating systems. Science 197: 215–223. 327542 10.1126/science.327542

[3] Trivers RL (1972) Parental investment and sexual selection. In Campbell B, ed., Sexual Selection and the Descent of Man, 1871–1971. Heinemann, London, U.K. 136–179.

[4] Wells KD (2007) The Ecology and Behaviour of Amphibians. University of Chicago Press, Chicago, Illinois, U.S.A.

[5] Shine R (1979) Sexual selection and sexual dimorphism in the Amphibia. Copeia 1979: 297–306.

[6] Han X, Fu J (2013) Does life history shape sexual dimorphism in Anurans? A comparative analysis. BMC Evol. Biol. 13: 27. 10.1186/1471-2148-13-27 PMC3570426 23368708

[7] Zheng Y, Li S, Fu J (2008) A phylogenetic analysis of the frog genera *Vibrissaphora* and *Leptobrachium*, and the correlated evolution of nuptial spine and reversed sexual size dimorphism. Mol Phylogenet Evol 46: 695–707. 17981478 10.1016/j.ympev.2007.09.019

[8] Duellman WE, Trueb L (1994) Biology of Amphibians. The Johns Hopkins University Pres, Baltimore.

[9] Wells KD (1977) The social behavior of anuran amphibians. Anim Behav 25: 666–693.

[10] Fei L, Ye C (1984) The biology and ecology of *Vibrissaphroa boringii* from Fanjing Mountain, Guizhou Province, China. Chinese J. Zool. 19: 1–4.

[11] Ye C, Fei L, Hu S (1993) Rare and Economic Amphibians of China. Sichuan Publishing House of Science and Technology, Chengdu.

[12] Zheng Y, Fu J (2007) Making a doughnut-shaped egg mass: Oviposition behaviour of *Vibrissaphora boringiae* (Anura: Megophryidae). Amphibia-Reptilia 28: 309–311.

[13] Zheng Y, Deng D, Li S, Fu J (2010) Aspects of the breeding biology of the Omei mustache toad (*Leptobrachium boringii*): polygamy and paternal care. Amphibia-Reptilia 31: 183–194.

[14] Vietes DR, Nieto-Roman S, Barluenga M, Palanca A, Vences M, et al (2004) Postmating clutch piracy in an Anuran amphibian. Nature 431: 305–308. 15372032 10.1038/nature02879

[15] Hudson CM, He X, Fu J (2010) Keratinized nuptial spines are used for male combat in the Emei Moustache Toad (*Leptobrachium boringii*). Asian Herp Res 2: 142–148.

[16] Ho TC, Lathrop A, Murphy RW, Orlov NL (1999) A redescription of *Vibrissaphora ailaonica* with a new record in Vietnam. Russ J Herpetol 6: 48–54.

[17] Ohler A, Teynié A, David P (2004) A green-eyed *Leptobrachium* (Anura: Megophryidae) from southern Laos. Raffles B Zool 52: 695–700.

[18] Bi K, Deng D, Crosby MKA, Fu J (2010) Characterization of microsatellite DNA markers in the Emei Moustache Toads (Leptobrachium boringii). Cons. Genet. 11: 1135–1137.

[19] Kalinowski ST, Taper ML, Marshall TC (2007) Revising how the computer program CERVUS accommodates genotyping error increases success in paternity assignment Mol Ecol. 16: 1099–1006. 10.1111/j.1365-294X.2007.03089.x 17305863

[20] Jones O, Wang J (2009) COLONY: a program for parentage and sibship inference from multilocus genotype data. Mol Ecol Res 10: 551–555. 10.1111/j.1755-0998.2009.02787.x 21565056

[21] Liu C, Hu S (1961) Tailless Amphibians of China. Science Press, Beijing, China.

[22] Orlov NL (2005) A new species of the genus *Vibrissaphora* Liu, 1945 (Anura: Megophryidae) from Mount Ngoc Linh (Kon Tum Province) and analysis of the extent of species overlap in the fauna of amphibians and reptiles of the North-West of Vietnam and Central Highlands. Russian J. Herpetol. 12: 17–38.

[23] Martins M, Pombal JP, Haddad CFB (1998) Escalated aggressive behaviour and facultative parental care in the nest building gladiator frog, *Hyla faber* . Amphibia-Reptilia 9: 49–60.

[24] Ryan MJ (1988) Energy, calling and selection. Amer Zool 28: 885–98.

[25] Schoener TW (1987) Time budgets and territory size: Some simultaneous optimization models for energy maximizers. Amer Zool 27: 259–291.

[26] Roldán M, Soler M (2011) Parental-care parasitism: How do unrelated offspring attain acceptance by foster parents? Behav Ecol 22: 679–691.

[27] Smith JM (1977) Parental investment: A prospective analysis. Anim Behav 25: 1–9.

[28] Ridley M, Rechten K (1981) Female sticklebacks prefer to spawn with males whose nests contain eggs. Behaviour 76: 152–161.

[29] Unger LM, Sargent RC (1988) Allopaternal care in the fathead minnow, *Pimephales promelas*: Females prefer males with eggs. Behav Ecol Sociobiol 23: 27–32.

[30] Zheng Y, Rao D, Murphy RW, Zeng X (2011) Reproductive behaviour and underwater calls in the Emei Moustache Toad, *Leptobrachium boringii* . Asian Herp Res 2: 199–215.

