# Supplementary material for: Male-Biased Sexual Size Dimorphism, Resource Defense Polygyny, and Multiple Paternity in the Emei Moustache Toad (Leptobrachium boringii)
Source: PLoS One. 2013 Jun 28;8(6):e67502. doi: 10.1371/journal.pone.0067502 (PMC3696078; doi:10.1371/journal.pone.0067502)
Supplement: Table S1 — Characterization of L. boringii microsatellite primers and tests for Hardy-Weinberg equilibrium. * = Significant P-value, deviating from Hardy-Weinberg equilibrium, NS = Non-significant following Bonferroni Correction (DOCX) [file pone.0067502.s001.docx]

| Locus | Repeat Motif | Primer Sequence 5'-3' | T_a_ | S | A | H_exp_ | H_obs_ | P-value | Bonferroni Correction | Null Allele Frequncy Estimate |
| --- | --- | --- | --- | --- | --- | --- | --- | --- | --- | --- |
| A6 | (GT)_21_ | F:TTCACATCACGCCGAGTGGCTGT | 58 | 332–390 | 7 | 0.7156 | 0.7119 | 0.385 | NS | -0.0149 |
| FJ809906 |  | R:CTATGCCTTCATCCTTGTGCCTC |  |  |  |  |  |  |  |  |
| B4 | (AG)_20_ | F:GACTACTCCTCTCCTGCC | 58 | 250–274 | 5 | 0.5921 | 0.5968 | 0.9752 | NS | -0.0123 |
| FJ809908 |  | R:ACCCAGGACTGCTTGACGAT |  |  |  |  |  |  |  |  |
| C3 | (GT)_19_ | F:CCTTAATGACATCTGCCCAGCT | 58 | 242–260 | 10 | 0.8264 | 0.9286 | 0.4281 | NS | -0.0647 |
| FJ809910 |  | R:CTCGAATTCTTAGCCCTCCT |  |  |  |  |  |  |  |  |
| C10 | (TATC)_17_ | F:GACACGGACTGAAACTCT | 58 | 224-252 | 8 | 0.7922 | 0.8871 | 0.2954 | NS | -0.0615 |
| FJ809912 |  | R:GATGGTCTTGCTTTCTGT |  |  |  |  |  |  |  |  |
| D5 | (AC)_14_AAA | F:ACCTGCCACATCATCCACAAAC | 58 | 274-298 | 9 | 0.8329 | 0.9344 | 0.0196* | NS | -0.066 |
| FJ809913 |  | R:GTATGGATTTAGAACGCAGCTC |  |  |  |  |  |  |  |  |
| D7 | (GT)_23_ | F:TTATTTGATCCGCTGACTCC | 58 | 186-204 | 7 | 0.7147 | 0.6721 | 0.8683 | NS | 0.0272 |
| FJ809914 |  | R:ACGCTTGGGTCACAGAACAT |  |  |  |  |  |  |  |  |
| F4 | (AC)_22_ | F:ACTACTGAAACGTCAGGT | 54 | 201-259 | 10 | 0.7802 | 0.5263 | 0.0165* | NS | 0.1848 |
| FJ809915 |  | R:CCAGACCATTCAGTGACATC |  |  |  |  |  |  |  |  |
