# Supplementary material for: Male-Biased Sexual Size Dimorphism, Resource Defense Polygyny, and Multiple Paternity in the Emei Moustache Toad (Leptobrachium boringii)
Source: PLoS One. 2013 Jun 28;8(6):e67502. doi: 10.1371/journal.pone.0067502 (PMC3696078; doi:10.1371/journal.pone.0067502)
Supplement: Table S2 — Genotypes of fathers and eggs from each egg mass. Alleles in bold do not belong to the primary father and represent maternal contribution, or genetic contribution from an unknown male. Alleles that are underlined are shared with putative secondary fathers that were located in the nest. Egg masses with a * display multiple paternity. (DOCX) [file pone.0067502.s002.docx]

| Nest # | Primary Father/ Egg Mass | Eggs | Locus C10 | Locus C3 | Locus B4 | Locus F4 | Locus D5 | Locus D7 | Locus A6 | Probability | Captured in nest? |
| --- | --- | --- | --- | --- | --- | --- | --- | --- | --- | --- | --- |
| 1 | **11-28** |  | 240/236 | 248/242 | 274/270 | 219/215 | 286/284 | 188/186 | ? | 93.3% | Yes |
|  | 1A | 8 | 240/240 (1) 240/236 (1) 240/**232** (1) 240/**224** (1) 236/**224** (3) 236/**232** (1) | **256**/**246** (2) **252**/**246** (3) **246**/**246** (1)  ? (2) | 274/270 (1) 274/**250** (3) 270/250 (4) | 219/215 (2) 215/215 (2)  ? (4) | **290**/286 (2) **290**/284 (1) 286/286 (2) 286/284 (1) 286/**274** (1)  ? (1) | **196**/186 (1) 188/188 (1) 188/186 (3) 186/186 (3) | **390**/**368** (2) **368**/**368** (4) **358**/**358** (1) **358**/**332** (1) |  |  |
| 2 | **11-20** |  | 240/236 | 260/252 | 270/270 | 219/219 | 288/286 | 198/196 | 358/358 | 90.7% | Yes |
|  | 2A | 9 | **248**/236 (1) 240/236 (1) 240/**224** (3) 236/**224** (4) | 260/**256** (1) 260/**248** (1) 260/**246** (1) 252/252 (1) 252/**246** (1)  ? (4) | **274**/270 (4) 270/270 (5) | **257**/**257** (1) **257**/**227** (1) **257**/219 (1) **227**/**227** (3)  ? (3) | 288/286 (1) 288/**282** (4) 286/286 (1) 286/**282** (1) 286/**274** (2) | 198/196 (7) 198/**186** (1)  ? (1) | **372**/358 (3) 358/358 (6) |  |  |
|  | **11-20** |  | 240/236 | 260/252 | 270/270 | 219/219 | 288/286 | 198/196 | 358/358 | 90.7% | Yes |
|  | 2B | 10 | **248**/236 (2) 240/240 (1) 240/236 (5) 236/236 (2) | 260/252 (1) 260/**248** (3) 252/**248** (1) 252/**246** (1)  ? (4) | 270/270 (10) | **257**/**257** (1) **257**/219 (1) **227**/**227** (6) 219/219 (2) | **290**/288 (1) **290**/286 (3) 288/288 (1) 288/286 (1) 286/**274** (3)  ? (1) | **200**/196 (1) 198/196 (5) 198/**186** (3)  ? (1) | **372**/258 (5) 358/358 (5) |  |  |
|  | **11-20** |  | 240/236 | 260/252 | 270/270 | 219/219 | 288/286 | 198/196 | 358/358 | 90.7% | Yes |
|  | 2C | 10 | **248**/240 (3) **248**/236 (4) 240/240 (1) 236/236 (2) | 260/260 (2) 260/252 (5) 260/**248** (2) 252/**248** (1) | 270/270 (10) | **257**/**257** (4) **257**/219 (1) **227**/**227** (2) **227**/219 (2)  ? (1) | 288/288 (5) 288/286 (4) 286/**274** (1) | 198/196 (3) 198/**186** (7) | 358/358 (10) |  |  |
|  |  |  |  |  |  |  |  |  |  |  |  |
|  | **11-28** |  | 240/236 | 248/242 | 274/270 | 219/215 | 286/284 | 188/186 | ? | 99.5% | Yes |
|  | 2D* | 10 | **248**/236 (2) 240/236 (1) 240/**232** (3) 236/236 (1) 236/**232** (1) 236/**224** (2) | 260/242 (2) **256**/**246** (1) **256**/242 (2) 248/242 (4)  ? (1) | 274/274 (3) 274/270 (3) 270/270 (4) | **257**/**257** (2) **257**/219 (3) **257**/215 (3) 219/215 (2) | **292**/284 (1) **290**/286 (1) **290**/284 (2) 286/**274** (4) 284/**274** (2) | **204**/186 (1) **198**/188 (1) **196**/188 (1) **196**/186 (1) 188/186 (4) 186/186 (2) | **390**/**390** (3) **390**/**358** (2) **390**/**332** (1) **358**/**358** (3) **358**/**332**( 1) |  |  |
|  | **11-20** |  | 240/236 | 260/252 | 270/270 | 219/219 | 288/286 | 198/196 | 358/358 | 90.7% | Yes |
|  | 2E | 10 | **248**/240 (3) **248**/236 (2) 240/236 (1) 236/236 (3) 236/**232** (1) | 260/260 (2) 260/**248** (3) 252/**248** (4) 260/252 (1) | 270/270 (10) | **257**/**257** (3) **257**/219 (1) **227**/**227** (1) **227**/219 (1) 219/219 (2) | 288/286 (1) 288/**274** (4) 286/**274** (5) | 198/198 (1) 196/196 (1) 198/**186** (4) 196/**186** (4) | 358/358 (10) |  |  |
| 3 | **11-03** |  | 240/232 | 252/246 | 274/270 | 257/219 | 286/284 | 196/186 | 390/358 | 99.8% | Yes |
|  | **2ndary father: 11-06** |  | 232/228 | 252/246 | 274/270 | 257/219 | 292/284 | 186/186 | 368/358 | 86.5% | Yes |
|  | 3A* | 10 | **252**/228 (1) 240/**236** (6) **236**/232 (2) 228/**224** (1) | 252/246 (5) 252/**248** (2) **248**/246 (2) 246/246 (1) | 274/274 (3) 274/270 (3) 270/270 (4) | 257/257 (2) 257/219 (2) 219/219 (5)  ? (1) | 292/**290** (1) **290**/284 (1) **288**/286 (2) **288**/284 (2) 286/**274** (4) | 196/186 (7) **188**/186 (1) 186/186 (2) | 390/358 (1) 358/358 (7) 358/**332** (2) |  |  |
|  | **11-06** |  | 232/228 | 252/246 | 274/270 | 257/219 | 292/284 | 186/186 | 368/358 | 82.1% | Yes |
|  | 3B | 8 | **252**/232 (3) **252**/228 (2) 228/**224** (3) | 252/252 (1) 252/246 (4) 246/246 (3) | 274/274 (5) 274/270 (2) 270/270 (1) | 257/257 (3) 257/219 (3) 219/219 (2) | **290**/**288** (3) **288**/284 (5) | **188**/186 (5) 186/186 (3) | 368/**332** (3) 358/**332** (5) |  |  |
| 4 | **11-31** |  | 248/240 | 252/246 | 274/270 | 219/219 | 288/274 | 198/186 | 368/358 | 87.7% | Yes |
|  | 4A | 10 | 248/248 (6) 248/240 (2) **244**/240 (2) | **258**/**254** (3) **258**/252 (1) **256**/252 (1) **256**/246 (1) **254**/246 (1)  ? (3) | 274 /274(2)  274/**250** (2) 270/270 (1) 270/**250** (2)  ? (3) | 219/219 (8) 219/**215** (2) | 286/274 (8) 274/274 (2) | 198/**196** (1) 198/186 (4) 186/186 (5) | 368/368 (7) 368/358 (2)  ? (1) |  |  |
|  |  |  |  |  |  |  |  |  |  |  |  |
| 6 | **11-13** |  | 244/224 | 252/246 | 274/270 | 259/259 | 288/284 | 196/186 | 358/332 | 86.6% | No |
|  | 6A | 10 | **248**/**248** (3) **248**/244 (7) | 252/252 (1) 252/246 (3) 246/246 (5)  ? (1) | 274/274 (6) 274/270 (4) | **219**/**219** (10) | **290**/**290** (2) **290**/284 (4) **290**/**274** (1) 284/**274** (3) | **198**/196 (5) **198**/186 (3) 196/186 (1) 186/186 (1) | 358/258 (5) 358/332 (4) 332/332 (1) |  |  |
|  | **11-03** |  | 240/232 | 252/246 | 274/270 | 257/219 | 286/284 | 196/186 | 390/358 | 90.7% | Yes |
|  | 6B | 10 | 240/232 (1) 240/**224** (4) 232/232 (3) 232/**224** (2) | **260**/252 (2) **260**/246 (1) **256**/252 (4) **256**/246 (1)  ? (2) | 274/**250** (4) 270/**250** (6) | **259**/259 (1) **259**/257 (2) 257/219 (6) 257/257 (1) | **290**/286 (4) **290**/284 (5)  ? (1) | 198/186 (7) 186/186 (3) | 390/390 (1) 390/358 (6) **368**/358 (3) |  |  |
|  | **11-03** |  | 240/232 | 252/246 | 274/270 | 257/219 | 286/284 | 196/186 | 390/358 | 96.2% | Yes |
|  | 6C | 5 | **252**/240 (2) **252**/232 (1) **248**/240 (1) 232/232 (1) | 260/246 (2) 252/246 (1)  ? (2) | 274/270 (3) 270/270 (2) | 257/219 (2) 219/219 (2)  ? (1) | **290**/284 (3) 286/**274** (1) 284/**274** (1) | 198/186 (3) 186/186 (2) | 358/358 (5) |  |  |
| 7 | **11-35** |  | 248/248 | 252/248 | 270/270 | 257/257 | 286/284 | 198/196 | 368/332 | 100% | Yes |
|  | 7A | 9 | 248/**236** (6) 248/**232** (3) | 252/248 (3) 248/248 (5)  ? (2) | **274**/270 (2) 270/270 (2) 270/**268** (4)  ? (1) | 257/257 (6) 257/**207** (3) | **290**/284 (1) **290**/**282** (2) 284/**282** (4) **282**/**282** (2) | 198/196 (8) 196/196 (1) | 368/358 (1) 368/332 (1) 358/332 (4)  ? (3) |  |  |
|  | **11-35** |  | 248/248 | 252/248 | 270/270 | 257/257 | 286/284 | 198/196 | 368/332 | 100% | Yes |
|  | 7B | 10 | 248/**236** (7) 248/**228** (1) 248/**232** (1)  ? (1) | 252/248 (3) 248/248 (4)  ? (3) | **274**/270 (5) 270/**268** (5) | 257/257 (3) 257/**207** (6)  ? (1) | **290**/284 (2) **290**/**282** (3) 284/**282** (2) **282**/**282** (3) | 198/198 (1) 198/196 (4) 196/196 (4)  ? (1) | **372**/368 (1) **372**/332 (2) 368/**358** (5) **358**/332 (2) |  |  |
|  |  |  |  |  |  |  |  |  |  |  |  |
| 8 | **11-08** |  | 236/232 | 260/248 | 270/250 | 219/219 | 288/284 | 198/198 | 368/358 | 90.1% | No |
|  | 8A | 8 | **248**/236 (5) **248**/232 (2)  ? (1) | **254**/248 (5) 248/**244** (2)  ? (1) | **274**/**274** (1) **274**/270 (3) 270/270 (4) | **257**/**209** (1) **227**/219 (1) 219/**209** (3)  ? (3) | **290**/288 (1) **290**/284 (3) 288/**282** (4) | 198/**196** (5) 198/**186** (1) **196**/**196** (1) **196**/**188** (1) **188**/**188** (2) | 368/368 (1) 368/**332** (3)  ? (4) |  |  |
| 11 | **Unknown** |  |  |  |  |  |  |  |  | N/A | N/A |
|  | 11A | 10 | 248/248 (2) 248/240 (2) 248/224 (6) | 260/248 (1) 252/252 (3) 252/248 (5) 248/244 (1) | 274/274 (5) 274/270 (4) 270/270 (1) | 257/257 (5) 257/219 (4) 219/219 (1) | 290/288 (4) 290/186 (1) 288/288 (2) 288/286 (3) | 204/188 (1) 198/188 (2) 198/186 (3) 188/188 (1) 188/186 (3) | 372/358 (6) 268/258 (2) 358/332 (2) |  |  |
|  | **Unknown** |  |  |  |  |  |  |  |  | N/A | N/A |
|  | 11B | 7 | 248/240 (5) 248/224 (2) | 260/248 (3) 258/254 (2) ? (2) | 274/270 (4) 270/270 (3) | 257/257 (1) 257/219 (3) 257/227 (3) | 292/290 (3) 292/288 (1) 290/290 (2) 290/288 (1) | 198/188 (1)  196/ 188 1)  188/188 (4)  186/186 (1) | 358/358 (3) 358/332 (4) |  |  |
| 12 | **11-17** |  | 248/224 | 260/248 | 270/250 | 259/219 | 288/284 | 198/186 | 372/358 | 99.6% | Yes |
|  | 12A | 7 | 248/224 (1) **236**/224 (2) 224/224 (3)  ? (1) | 260/248 (4) 248/**246** (2) **246**/**246** (1) | 270/270 (6) 270/250 (1) | 259/**227** (3) 259/**209** (3) 219/219 (1) | 290/288 (3) 288/**274** (1) **284**/**274** (3) | 198/198 (2) 198/186 (2) 186/186 (3) | 372/358 (1) 358/358 (6) |  |  |
|  | **11-17** |  | 248/224 | 260/248 | 270/250 | 259/219 | 288/284 | 198/186 | 372/358 | 99.6% | Yes |
|  | 12B | 7 | **252**/248 (5) 248/**236** (1) **236**/224 (1) | 260/**252** (4) 260/**254** (2) **252**/**252** (1) | **274**/250 (2) 270/250 (3) 250/250 (2) | 259/**257** (1) 259/219 (1) **257**/219 (2)  219/219 (1)  ? (2) | **290**/288 (4) 288/**286** (2) 284/284 (1) | 198/198 (4) 198/186 (3) | 372/372 (2) 372/**332** (4) **332**/**332** (1) |  |  |
|  | **11-17** |  | 248/224 | 260/248 | 270/250 | 259/219 | 288/284 | 198/186 | 372/358 | 97.7% | Yes |
|  | 12C | 10 | 248/**232** (2) 248/**228** (2) **232**/224 (3) **228**/224 (3) | 260/248 (1) 260/**246** (1) **258**/**246** (3) **254**/**246** (1) 248/**246**(1) **246**/**246** (2)  ? (1) | **274**/270 (5) **274**/250 (5) | 259/259 (2) 259/219 (5) 219/219 (1) 219/**201** (1) | 288/286 (3) 288/**282** (1) **286**/284 (2) 284/**282** (4) | 198/198 (5) 198/186 (5) | 372/**362** (3) 372/358 (1) **362**/358 (3) 358/358 (1) |  |  |
| 13 | **11-29** |  | 248/248 | 260/256 | 270/250 | 257/209 | 294/284 | 186/186 | 372/372 | 95.7% | Yes |
|  | 13A | 10 | 248/248 (2) 248/**244** (1) 248/**236** (2) 248/**232** (5) | 260/**252** (7) **252**/**246** (2)  ? (1) | **274**/250 (1) 270/270 (5) 270/250 (4) | 257/**219** (2) 257/209 (4) **219**/**219** (2) **219**/209 (1) 209/209 (1) | 292/284 (4) 292/**274** (3) 284/**274** (3) | **198**/**198** (4) **198**/**196** (1) **198**/186 (5) | 372/372 (2) 372/**358** (4) **358**/**358** (4) |  |  |
| 14 | **11-06** |  | 232/228 | 252/246 | 274/270 | 257/219 | 292/284 | 186/186 | 368/358 | 100% | Yes |
|  | 14A | 10 | **248**/228 (2) 232/232 (5) 232/228 (3) | 252/**244** (5) **244**/**244** (5) | 274/270 (2) 274/**250** (1) 270/270 (1) 270/**250** (6) | 257/257 (5) 257/219 (3) 219/219 (2) | 292/**290** (2) 292/**274** (2) **290**/284 (3) 284/**274** (3) | 188/186 (3) 186/186 (7) | 372/358 (5) 358/358 (5) |  |  |
| 15 | **11-14** |  | 240/232 | 252/248 | 274/270 | 257/219 | 288/284 | 196/186 | 372/358 | 97.6% | Yes |
|  | 15A | 8 | 240/**236** (3) **236**/232 (4) 232/232 (1) | 252/**244** (4) **246**/**244** (2) **246**/**242** (2) | 274/270 (4) 270/270 (4) | 257/219 (2) 257/**207** (3) 219/**207** (3) | **290**/284 (4) **290**/288 (4) | **198**/196 (2) **198**/186 (5) 186/186 (1) | 372/372 (8) |  |  |
| 16 | **11-21** |  | 248/240 | 260/252 | 250/250 | 219/209 | 284/284 | 198/186 | 368/358 | 56.6% | No |
|  | 16A | 10 | 248/240 (2) 248/**236** (1) 240/240 (4) 240/**236** (3) | 252/252 (1) 252/**244** (6) **246**/**242** (1) **244**/**244** (2) | **274**/**270** (1) **270**/**270** (5) **270**/250 (4) | **257**/219 (2) **257**/209 (3) **257**/**207** (2) 209/**207** (3) | **294**/**290** (4) **294**/**286** (2) **290**/284 (2) **286**/284 (2) | **196**/186 (3) 186/186 (7) | **390**/258 (7) 258/258 (3) |  |  |
| N/A (Trial) | **11-54** |  | 248/236 | 252/246 | 270/270 | 219/215 | 292/284 | 190/186 | 368/362 | 100% | N/A |
|  | 5455A | 9 | 248/236 (2) 248/**232** (3) 236/236 (2) 236/**232** (1) | **260**/252 (2) **260**/246 (3) 252/**244** (2) 246/**244** (1)  ? (1) | 270/270 (5) 270/**250** (4) | 219/219 (3) 219/**201** (2) 215/**201** (3)  ? (1) | 292/274 (3) **290**/284 (2) 284/**274** (4) | **198**/190 (3) **198**/186 (4)  ? (2) | **390**/368 (2) **390**/362 (3) 368/362 (4) |  |  |
